# Physically Constrained Dual-Branch Front-End Optimization for DDH-Oriented Surgical Robot Navigation

**DOI:** 10.3390/bioengineering13080895

**Published:** 2026-08-03

**Authors:** Jiabao Li, Ming Zhu, Chengjun Wang, Kang Xie, Shaoyue Wang, Ziyang Wang, Dongdong Ye

**Affiliations:** 1 School of Artificial Intelligence, Anhui Polytechnic University, Wuhu 241000, China; lijiabao@ahpu.edu.cn (J.L.); zm355693@163.com (M.Z.); wangchengjun@ahpu.edu.cn (C.W.); 2 Anhui Provincial Key Laboratory of Intelligent Diagnosis and Precision Treatment for Pediatric Bone Diseases, Hefei 230000, China; 19900130@163.com; 3 School of Computer Science and Digital Technologies, Aston University, Birmingham B4 7ET, UK; 2022307566@aust.edu.cn (S.W.); z.wang47@aston.ac.uk (Z.W.); 4 Aviation Industry Corporation Huadong Photoelectric Co., Ltd., Wuhu 241003, China

**Keywords:** developmental dysplasia of the hip, surgical robot navigation, physically constrained learning, dual-branch neural network, target-representation optimization, robot motion planning

## Abstract

Surgical robot navigation in a restricted pelvic workspace requires accurate target localization, directional consistency and robot-feasible execution. This study proposes a physically constrained dual-branch front-end network (PCD-Net) for DDH-oriented navigation using public CT-derived pelvis geometries. PCD-Net maps a 21-dimensional input comprising the target geometry, current joint state, workspace bounds and constraint parameters to a base-frame target position, principal insertion direction and seven-joint correction vector for MoveIt2 and OMPL planning. Training combines supervised pretraining with direction consistency, joint limit, correction magnitude and workspace gap penalties. Evaluation comprised 15 complete simulation trials per method and target sequence-level fivefold cross-validation of 999 samples from five complete sequences. PCD-Net achieved a position error of 0.736±0.202 mm, a direction error of 0.428±0.130°, a planning time of 0.0162±0.0040 s and successful execution in all 15 trials. In cross-validation, the complete constraint setting produced the lowest joint correction MAE (0.755±0.034 rad) and temporal correction variation (3.270±0.589 rad) while maintaining sub-millimeter position and sub-degree direction errors. Removing the joint limit penalty increased the violation rate by 42.35%. These results support PCD-Net as a lightweight, planner-compatible front end that balances geometric accuracy and joint-level feasibility. All evidence is simulation-based; phantom experiments, physical robot validation and evaluation using clinically characterized DDH cases remain necessary before surgical translation.

## 1. Introduction

Developmental dysplasia of the hip (DDH) presents complex pelvic geometry and motivates accurate, reproducible image-guided planning. Robotic arm-assisted hip procedures have demonstrated the importance of spatial registration, component positioning and controlled instrument orientation in dysplastic and anatomically challenging hips [1,2,3,4]. A navigation target should therefore include not only a spatial point but also an insertion direction, transformed coordinate representation, robot state and local workspace constraints.

Computer-assisted surgical systems commonly separate anatomical target definition from robot trajectory generation [5,6,7,8]. This modular architecture is practical, but a geometrically meaningful anatomical target may remain difficult to execute after transformation into the robot base frame. Sampling-based and trajectory optimization planners generate collision-free motion after receiving a goal representation [9,10,11,12]; they do not necessarily refine the upstream target according to task-specific directional and joint feasibility requirements.

Learning-based front ends can model nonlinear relations among target geometry, coordinate transformations and robot states, but purely data-driven models may produce joint limit violations or unstable correction outputs. Physics-informed and physics-guided learning incorporate governing relations or operational feasibility penalties into training [13,14,15]. Related robotic studies have addressed force estimation, dynamics identification, friction modeling, torque estimation and sim-to-real transfer [16,17,18,19,20]. CRAB-EDM further illustrates how physical mechanisms and temporally structured control can be coordinated in a robotic system [21], although its underwater application differs from surgical navigation.

Classical physics-informed neural networks are typically formulated around governing-equation residuals and collocation. The present method instead embeds operational robot and workspace constraints in a supervised dual-branch network. It is therefore termed the physically constrained dual-branch front-end network. (PCD-Net). PCD-Net refines a position–direction representation and predicts a joint correction vector before conventional planning; it does not replace MoveIt2, OMPL or low-level robot control.

Public CT-derived anatomy provides geometry for simulation but does not constitute clinical DDH validation. Pelvis and hip-related structures were prepared from a screened subset of the TotalSegmentator dataset [22] using established medical image and surface reconstruction tools [23,24]. The resulting STL models were used for DDH-oriented task construction and collision representation and not as a clinically confirmed pediatric DDH cohort.

**Figure 1:**
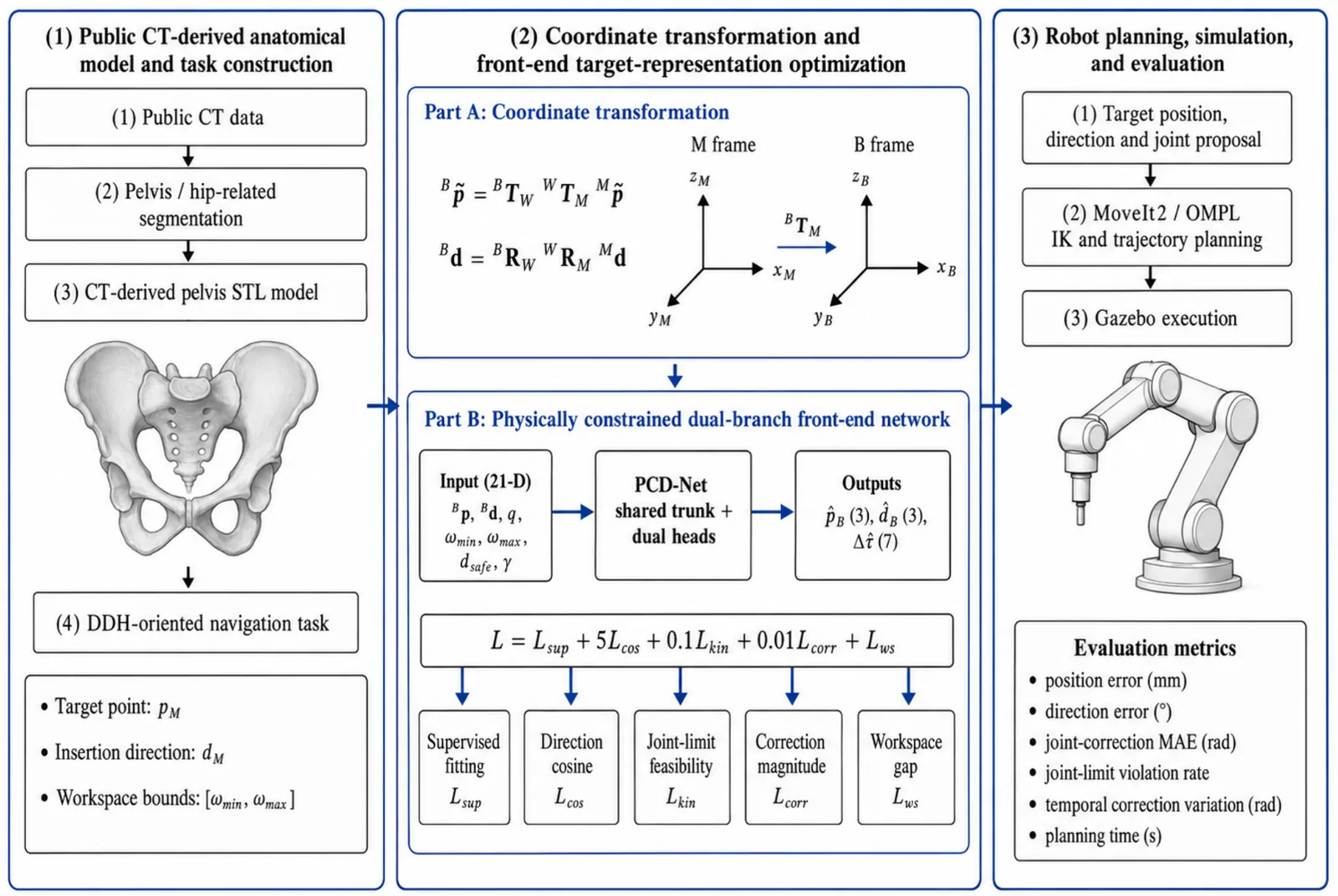
Overall framework. Public CT-derived pelvis geometry defines the target position, insertion direction and workspace bounds. PCD-Net predicts a base-frame position–direction representation and seven-joint correction vector before MoveIt2 or OMPL planning and Gazebo evaluation.

The contributions are fourfold: (1) a coupled target position, insertion direction and joint correction formulation for a MoveIt2-compatible front end; (2) a fully specified 21-input, dual-branch architecture with operational robot and workspace penalties; (3) pipeline-level evaluation and target sequence-level fivefold analysis using 25 independently trained models and (4) an explicit workspace margin feasibility condition that prevents incompatible safety gap settings from being interpreted as evidence of navigation safety. The overall framework is illustrated in Figure 1.

## 2. Materials and Methods

### 2.1. Study Scope and Anatomical Model Preparation

This study was designed as a simulation-based methodological investigation. Public CT data were obtained from the TotalSegmentator dataset, which provides automatically segmented anatomical structures from whole-body CT images [22]. Cases were screened according to the completeness of the pelvic anatomy, bilateral femoral heads and acetabular regions. Forty-one cases satisfying these anatomical completeness criteria were retained for model preparation.

The pelvis and hip-related segmentations were inspected and processed using 3D Slicer (https://www.slicer.org, accessed on 23 July 2026) [23]. Surface meshes were generated from the volumetric segmentations using a marching cubes-based reconstruction procedure [24], and the resulting models were exported in STL format. The reconstructed surfaces were used to define anatomical task geometry and to construct collision representations in the simulation environment.

The retained CT cases were not clinically confirmed as developmental dysplasia of the hip and were not treated as independent clinical outcome units. Their role was to provide anatomically variable pelvic geometries for DDH-oriented navigation task construction and simulation. Accordingly, model development and evaluation were performed at the simulated target sequence level rather than at the patient outcome level.

### 2.2. Coordinate Frames and Navigation Task

Four coordinate frames were considered: the anatomical model frame $F_{M}$, the simulation or world frame $F_{W}$, the robot base frame $F_{B}$ and the end-effector frame $F_{E}$. The anatomical target point and insertion direction were initially defined in $F_{M}$ and then deterministically transformed into $F_{B}$ before being provided to the front-end network.

For a target point expressed in homogeneous coordinates, the frame transformation was defined as follows:

(1) p˜B=TWBTMWp˜M,

where p˜M and p˜B denote the target point coordinates in the anatomical model frame and robot base frame, respectively, and TWB and TMW are rigid homogeneous transformation matrices.

Because the insertion direction is a vector rather than a point, only the rotational components of the transformations were applied:

(2) d¯B=RWBRMWdM,dB=d¯Bd¯B2+ϵ,

where dM and dB denote the insertion-direction vectors in the anatomical model frame and robot base frame, respectively, and ϵ is a small positive constant introduced for numerical stability.

**Figure 2:**
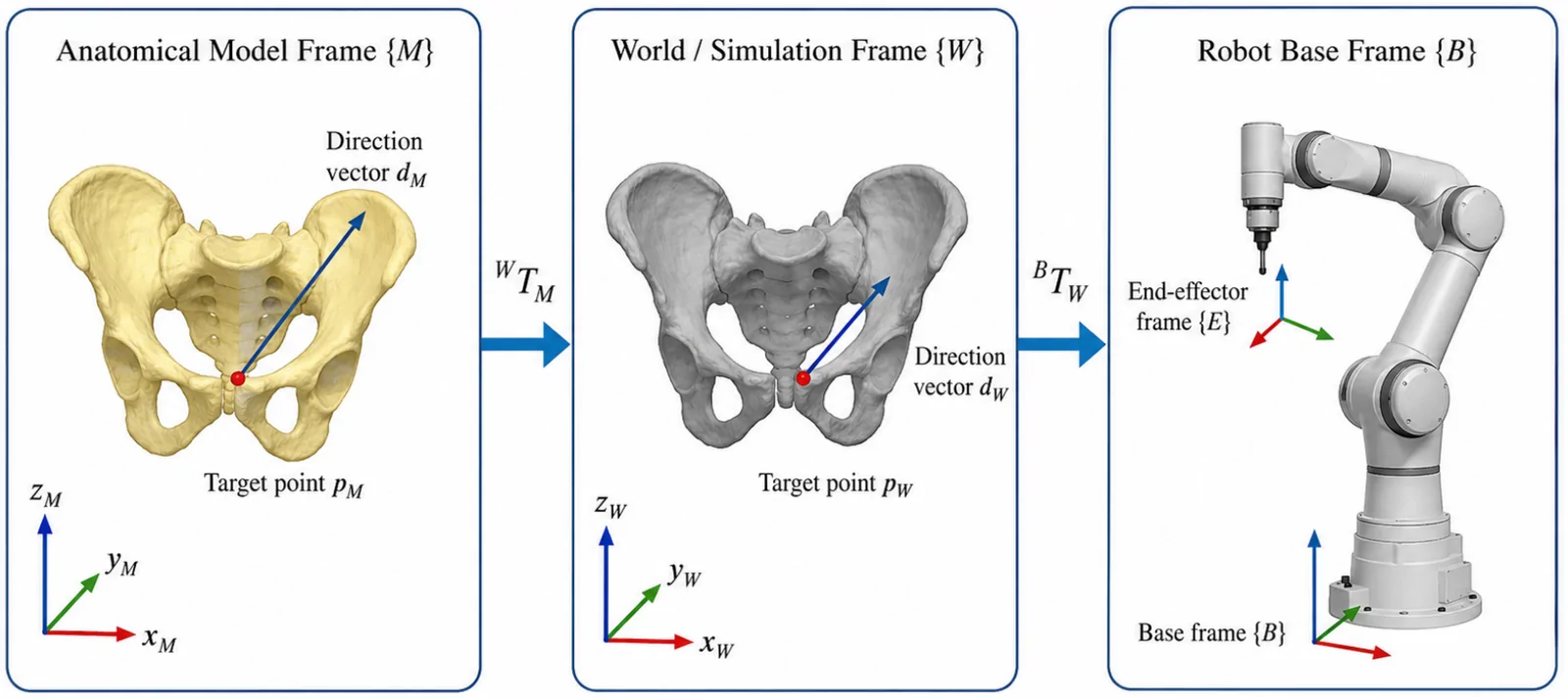
Coordinate-frame mapping of the target point and insertion direction. The anatomical target defined in the model frame is transformed through the simulation or world frame into the robot base frame before front-end optimization and robot planning.

The navigation target was represented by a base frame position and a principal tool axis direction rather than by a complete homogeneous end effector transformation (Figure 2). The remaining roll degree of freedom was assigned by the deterministic planner-side goal construction convention. This formulation intentionally restricts the learned output to the translational target and principal insertion axis required by the downstream planner, thereby avoiding unconstrained regression of a complete rotation matrix.

For navigation sample *i*, the 21-dimensional network input was defined as follows:

(3) xi=piB,diB,qi,ωmin,i,ωmax,i,dsafe,i,γi,

where piB∈R3 and diB∈R3 are the transformed target position and unit insertion direction, $q_{i}\in R^{7}$ is the current robot joint state, $\omega_{\min,i},\omega_{\max,i}\in R^{3}$ are the axis-aligned workspace bounds expressed in the robot base frame, $d_{\mathrm{safe},i}$ is the workspace-gap parameter and $\gamma_{i}$ is the joint-correction regularization factor. The resulting dimensionality is 3+3+7+3+3+1+1=21.

The network output was

(4) $${\hat{y}}_{i}=\left[{\hat{p}}_{B,i},{\hat{d}}_{B,i},\Delta {\hat{\tau }}_{i}\right],\;\Delta {\hat{\tau }}_{i}\in R^{7},$$

where ${\hat{p}}_{B,i}$ is the predicted base-frame target position, ${\hat{d}}_{B,i}$ is the predicted principal insertion direction and $\Delta {\hat{\tau }}_{i}$ is the predicted seven-joint correction vector.

#### Planner-Side Label Generation and Inference Interface

The supervised position–direction targets were obtained from planner-executable base-frame target representations retained from successful simulation records. For each accepted record, MoveIt2 generated an inverse-kinematics solution $q_{\mathrm{goal}}$ under the common robot model, joint limits and collision representation. The corresponding joint correction label was calculated as follows:

(5) $$\Delta \tau =q_{\mathrm{goal}}-q_{\mathrm{current}}.$$

Records with unsuccessful inverse kinematics or trajectory planning were excluded before the target sequence partition was constructed. No loss-based filtering or constraint-dependent sample removal was performed after the training, validation and test sequences were assigned.

During inference, ${\hat{p}}_{B}$ and ${\hat{d}}_{B}$ define the Cartesian position–direction target, while

(6) $${\hat{q}}_{\mathrm{proposal}}=q_{\mathrm{current}}+\Delta \hat{\tau }$$

provides a feasibility-conditioned joint proposal to the planner interface. MoveIt2 remains responsible for roll assignment, inverse kinematics, joint-limit checking, collision checking and OMPL or RRTConnect trajectory generation.

### 2.3. PCD-Net Architecture

**Figure 3:**
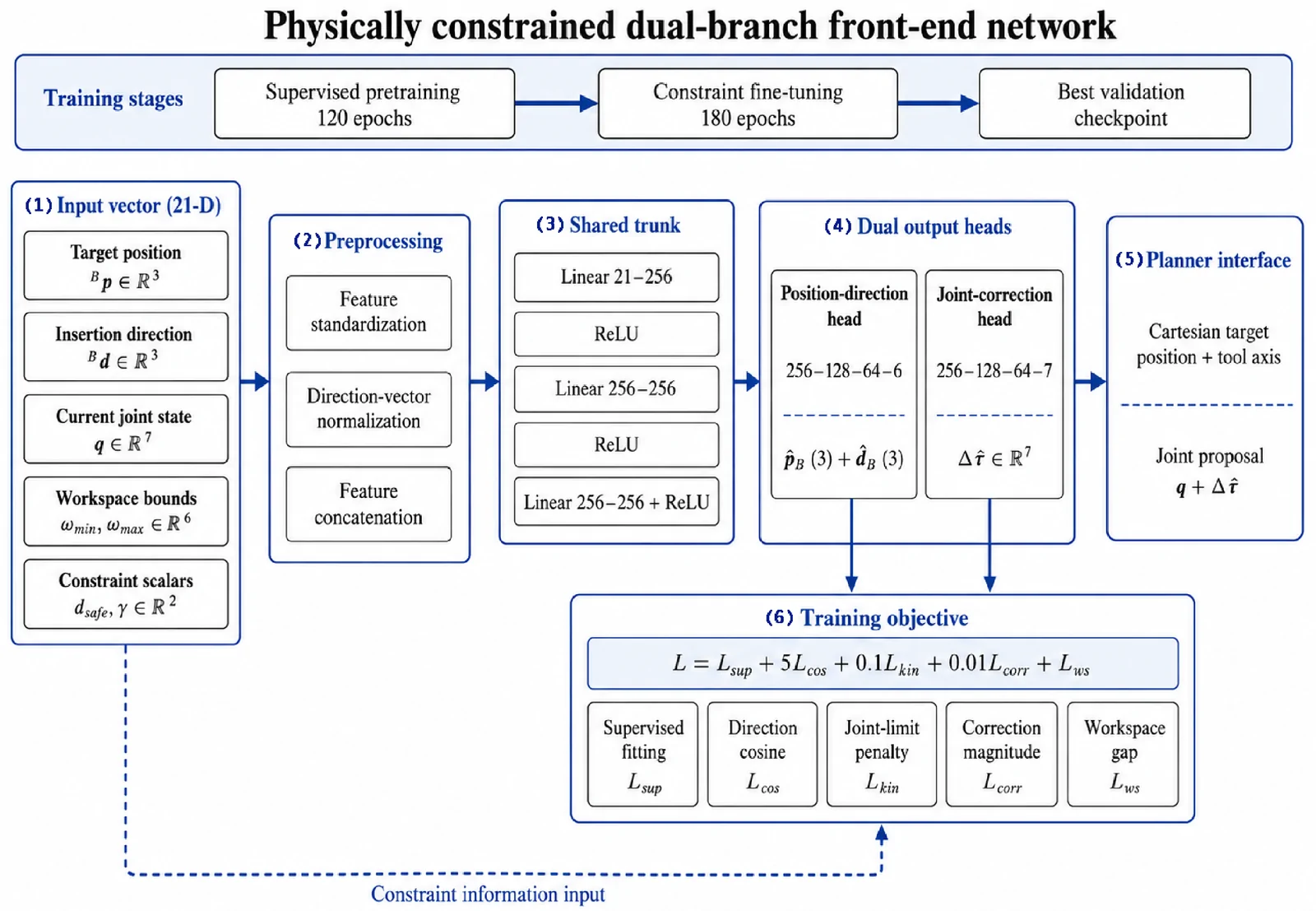
PCD-Net architecture and two-stage training strategy. A shared trunk processes the 21-dimensional input, and two task-specific heads predict the base-frame position–direction target and seven-joint correction vector. The outputs are passed to the planner interface and optimized using supervised and physically constrained loss terms.

PCD-Net consists of a shared feature trunk and two task-specific output heads. For each cross-validation fold, scalar inputs and supervised outputs were standardized using statistics estimated from the training subset, while insertion-direction vectors were normalized to the unit length. The shared trunk contains three 256-unit fully connected layers with ReLU activations. The position–direction head uses two hidden layers of 128 and 64 units to produce six outputs, whereas the joint correction head uses the same hidden dimensions to produce seven outputs. The complete network contains 220,365 trainable parameters. Its main specifications are summarized in Table 1 and illustrated in Figure 3.

### 2.4. Supervised and Constraint Losses

For a mini-batch containing *N* samples, the supervised fitting loss was defined as follows:

(7) $$L_{\sup}=\frac{1}{N}\sum_{i=1}^{N}\left[\lambda_{p}{∥{\hat{p}}_{B,i}^{\;s}-p_{B,i}^{s}∥}_{2}^{2}+\lambda_{d,s}{∥{\hat{d}}_{B,i}^{\;s}-d_{B,i}^{s}∥}_{2}^{2}+\lambda_{\tau }{∥\Delta {\hat{\tau }}_{i}^{\;s}-\Delta \tau_{i}^{s}∥}_{2}^{2}\right],$$

where the superscript *s* denotes standardized variables. The supervised weights were $\lambda_{p}=1.0$, $\lambda_{d,s}=0.2$ and $\lambda_{\tau }=1.0$.

Directional consistency was imposed using

(8) $$L_{\cos}=\frac{1}{N}\sum_{i=1}^{N}\left(1-\frac{{\hat{d}}_{B,i}^{⊤}d_{B,i}}{{∥{\hat{d}}_{B,i}∥}_{2}{∥d_{B,i}∥}_{2}+\epsilon }\right),$$

where ϵ is a small positive constant for numerical stability.

For the predicted joint proposal

(9) $${\hat{q}}_{\mathrm{goal},i}=q_{i}+\Delta {\hat{\tau }}_{i},$$

the joint limit penalty was

(10) $$L_{\mathrm{kin}}=\frac{1}{N}\sum_{i=1}^{N}\left[{∥\mathrm{ReLU}\left(q_{\min}-{\hat{q}}_{\mathrm{goal},i}\right)∥}_{2}^{2}+{∥\mathrm{ReLU}\left({\hat{q}}_{\mathrm{goal},i}-q_{\max}\right)∥}_{2}^{2}\right],$$

where $q_{\min}$ and $q_{\max}$ denote the robot joint limits.

The joint correction magnitude was regularized by

(11) $$L_{\mathrm{corr}}=\frac{1}{N}\sum_{i=1}^{N}\gamma_{i}{∥\Delta {\hat{\tau }}_{i}∥}_{2}^{2},$$

where $\gamma_{i}$ controls the correction penalty.

The axis-aligned workspace-gap loss was

(12) $$L_{\mathrm{ws}}=\frac{1}{N}\sum_{i=1}^{N}[{∥\mathrm{ReLU}\left(d_{\mathrm{safe},i}1-\left({\hat{p}}_{B,i}-\omega_{\min,i}\right)\right)∥}_{2}^{2}\;\;\;\;\;\;+{∥\mathrm{ReLU}\left(d_{\mathrm{safe},i}1-\left(\omega_{\max,i}-{\hat{p}}_{B,i}\right)\right)∥}_{2}^{2}],$$

where $1\in R^{3}$ distributes the scalar safety-gap parameter across the three workspace axes.

The complete constraint fine-tuning objective was

(13) $$L=L_{\sup}+5.0L_{\cos}+0.1L_{\mathrm{kin}}+0.01L_{\mathrm{corr}}+1.0L_{\mathrm{ws}}.$$

#### Constraint-Weight Sensitivity Assessment

The operational contribution of each constraint term was evaluated using a one-at-a-time zero-weight analysis. The coefficient of $L_{\mathrm{kin}}$, $L_{\cos}$, $L_{\mathrm{corr}}$ or $L_{\mathrm{ws}}$ was set to zero while all other training settings remained unchanged. These configurations correspond to A1–A4, whereas A5 denotes the complete objective. This analysis evaluates dependence on individual constraint terms but does not imply global optimality of the selected nonzero weights.

### 2.5. Training Protocol and Target Sequence Cross-Validation

The model development dataset contained 999 sequential samples from five complete target sequences, denoted as T01–T05. Cross-validation was performed at the complete sequence level rather than at the individual frame level. In each fold, three sequences were used for training, with one for validation and one for testing, preventing adjacent samples from the same sequence from appearing in both the development and test subsets.

Five constraint configurations were trained in each fold, producing 25 independently trained models. All models used the same architecture, sequence partitions and optimization protocol. Training employed Adam with a learning rate of $1\times 10^{-3}$, a batch size of 64 and a fixed random seed of 42. Supervised pretraining was conducted for 120 epochs, followed by 180 epochs of constraint fine-tuning. The checkpoint with the lowest validation total loss during fine-tuning was retained for evaluation. The implementation environment and computational configuration are summarized in Table 2.

### 2.6. Simulation Platform, Comparison Groups and Evaluation Metrics

**Figure 4:**
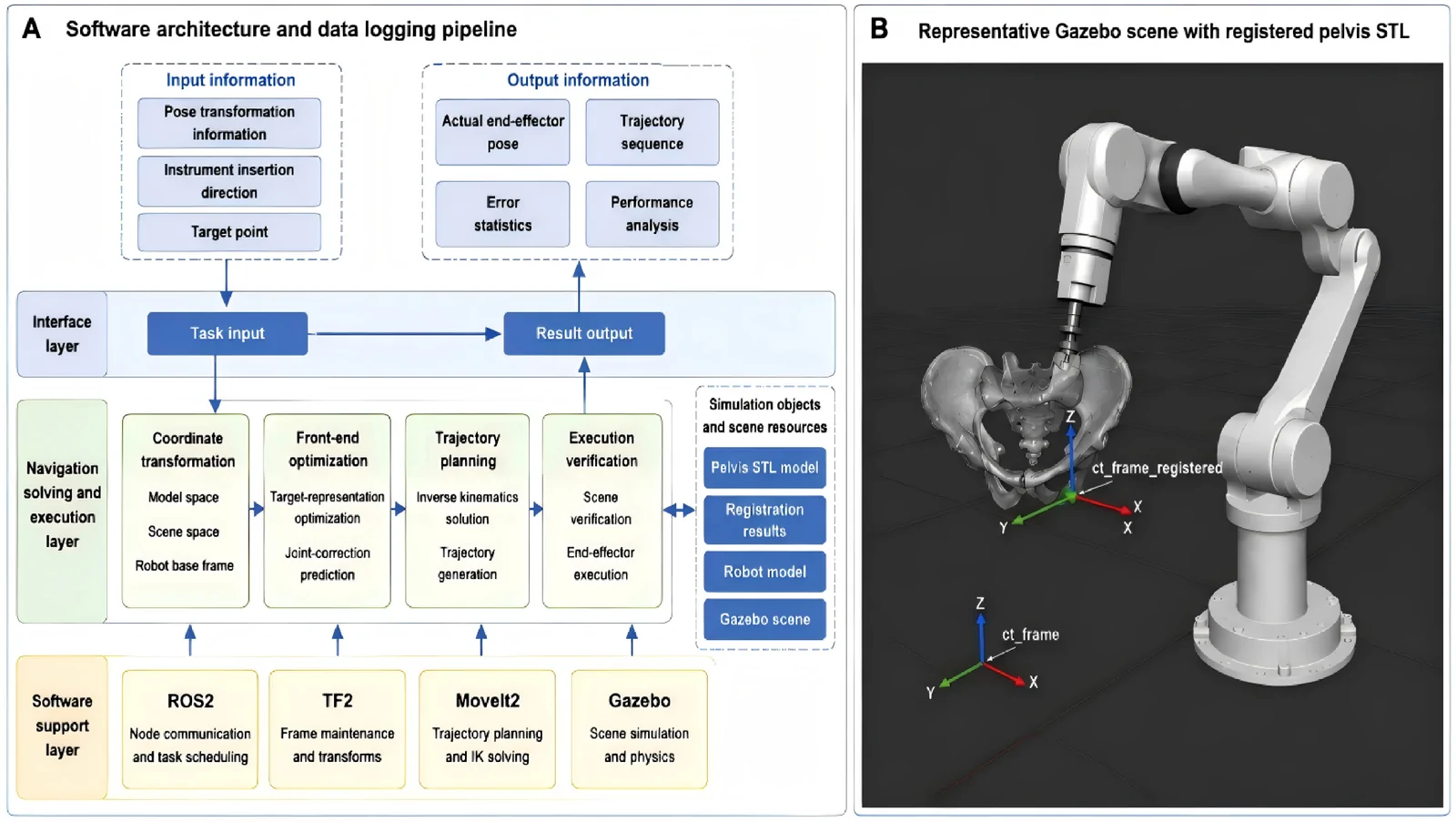
ROS2–MoveIt2–Gazebo simulation platform. (**A**) Software architecture and data processing pipeline. (**B**) Representative Gazebo scene containing the robot and registered pelvis model.

The simulation platform (Figure 4) integrated ROS2 Humble (Humble Hawksbill, https://docs.ros.org/en/humble/, accessed on 23 July 2026), TF2 (bundled with ROS2 Humble), MoveIt2 (https://moveit.ai, accessed on 23 July 2026), OMPL (https://ompl.kavrakilab.org, accessed on 23 July 2026) and Gazebo 11.14.0. ROS2 handled task communication, TF2 maintained coordinate–frame relations, MoveIt2 performed goal construction, inverse kinematics and collision checking, OMPL and RRTConnect (RRTConnect is a sampling-based planner implemented within OMPL) generated trajectories, and Gazebo provided simulated execution [25,26,27,28,29].

The pipeline-level comparison included five methods: G1, standard MoveIt2 inverse-kinematics goal projection with RRTConnect; G2, an unconstrained neural baseline; G3-NP, the dual-branch network without operational constraint penalties; G3-NR, the network without correction-magnitude regularization and the complete PCD-Net. Each method was evaluated in 15 complete simulation trials using the same robot model, collision representation, downstream planner and evaluation protocol.

Pipeline-level outcomes included planning success, position error, direction error and planning time. Position error was calculated as the Euclidean distance between the achieved and reference target positions and is reported in millimeters. Direction error was calculated as the angular difference between the achieved and reference principal insertion directions and is reported in degrees. Planning time was measured from planner request submission to trajectory response generation and is reported in seconds.

The target sequence-level fivefold analysis used the constraint configurations listed in Table 3 and additionally evaluated the joint correction mean absolute error (MAE), joint limit violation rate and temporal correction variation. The joint correction MAE was averaged across the seven robot joints and is reported in radians. The joint limit violation rate was defined as the proportion of predicted joint proposals exceeding at least one predefined joint limit. For a held-out sequence containing *K* ordered samples, temporal correction variation was defined as follows:

(14) $$V_{\Delta \tau }=\frac{1}{K-1}\sum_{k=1}^{K-1}{∥\Delta {\hat{\tau }}_{k+1}-\Delta {\hat{\tau }}_{k}∥}_{2},$$

and is reported in radians. Lower values indicate better performance for all continuous error and variation metrics.

Results are reported as the mean ± SD. For the five held-out target sequences, 95% confidence intervals were calculated using the Student-*t* distribution with four degrees of freedom. Exact two-sided sign-flip tests were used only to describe fold-wise directional consistency. No statistical significance claim was made because the number of independent target sequences was five.

## 3. Results

### 3.1. Pipeline-Level Comparison

**Figure 5:**
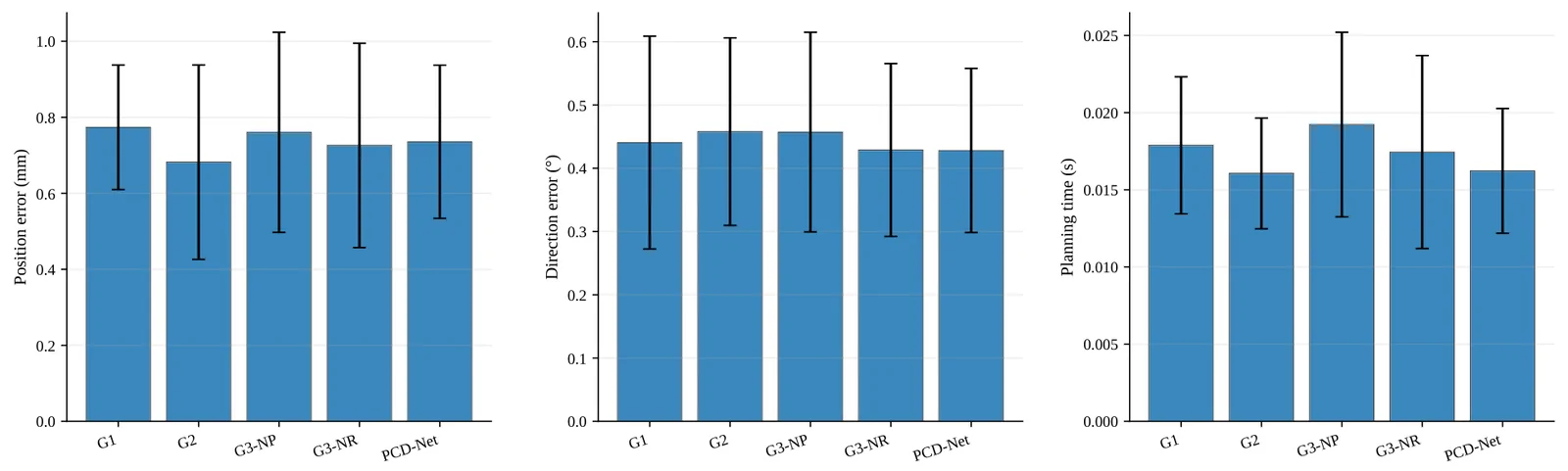
Pipeline-level comparison. Bars show means and error bars show SD across 15 complete simulation trials per method. G1: standard IK and RRTConnect; G2: unconstrained neural network; G3-NP: dual branch without constraint penalties; G3-NR: without correction magnitude regularization; PCD-Net: complete model.

All five methods completed 15/15 simulation trials. Table 4 and Figure 5 summarize the results. PCD-Net achieved a 0.736±0.202 mm position error, 0.428±0.130° direction error and 0.0162±0.0040 s planning time. Relative to the standard IK and RRTConnect, these values represented descriptive reductions of 4.93%, 2.84% and 9.26%, respectively. Relative to the unconstrained neural baseline, PCD-Net reduced the direction error by 6.49% and had a comparable planning time, whereas the unconstrained model retained the lowest mean position error. The result therefore supports a direction control and feasibility-oriented interpretation rather than universal superiority across every isolated metric.

### 3.2. Target Sequence-Level Fivefold Analysis

**Figure 6:**
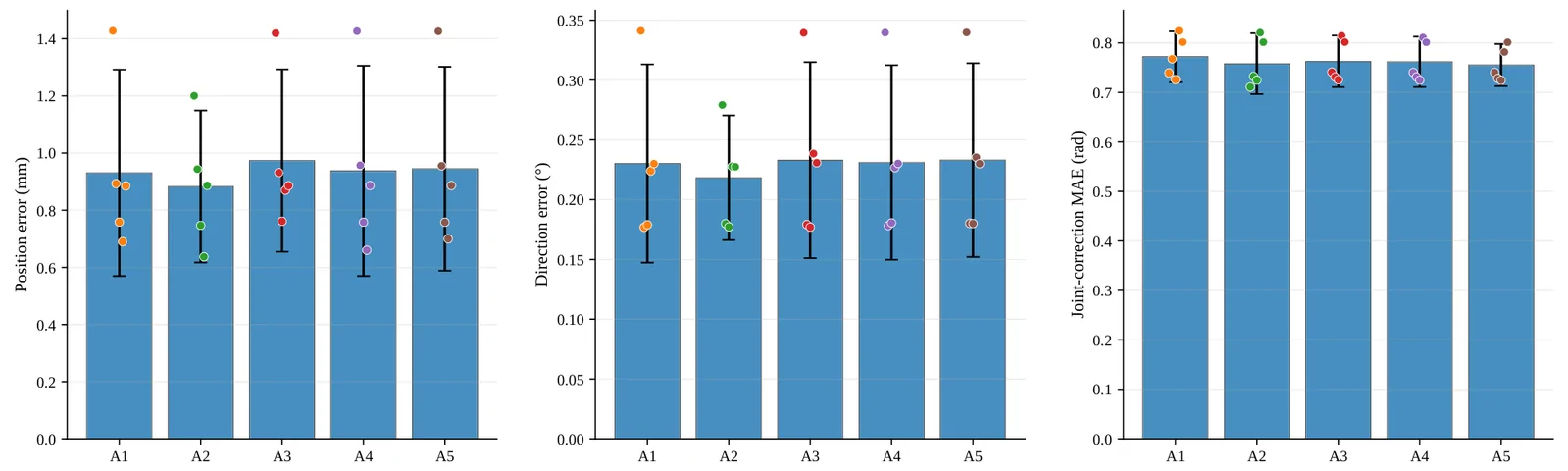
Target sequence-level fivefold sensitivity analysis. Bars show fold means, error bars show 95% confidence intervals, and points show the five held-out target sequence results.

All 25 planned training runs completed. Table 5 summarizes the target sequence-level fivefold results, and Figure 6 shows the fold means and 95% confidence intervals with the five held-out sequence results overlaid. A2 produced the lowest mean position and direction errors. The complete model A5 maintained a sub-millimeter position error (0.945±0.287 mm) and sub-degree direction error (0.233±0.065°) while producing the lowest joint correction MAE (0.755±0.034 rad). The between-fold coefficient of variation for the A5 joint correction MAE was 4.54%, the lowest among the five settings.

### 3.3. Zero-Weight Sensitivity and Multi-Objective Trade-Off

Removing the joint limit term increased the mean violation rate from 0.071 in A5 to 0.101 in A1, a 42.35% increase relative to the complete model. Removing correction-magnitude regularization increased the joint correction MAE from 0.755 to 0.763 rad and temporal correction variation from 3.270 to 3.327 rad. A5 yielded lower values than A3 for both outcomes in all five held-out target sequences; the exact two-sided sign-flip probability for a 5/5 directional result was 0.0625. These fold-wise effects are reported as consistency evidence rather than inferential significance.

**Figure 7:**
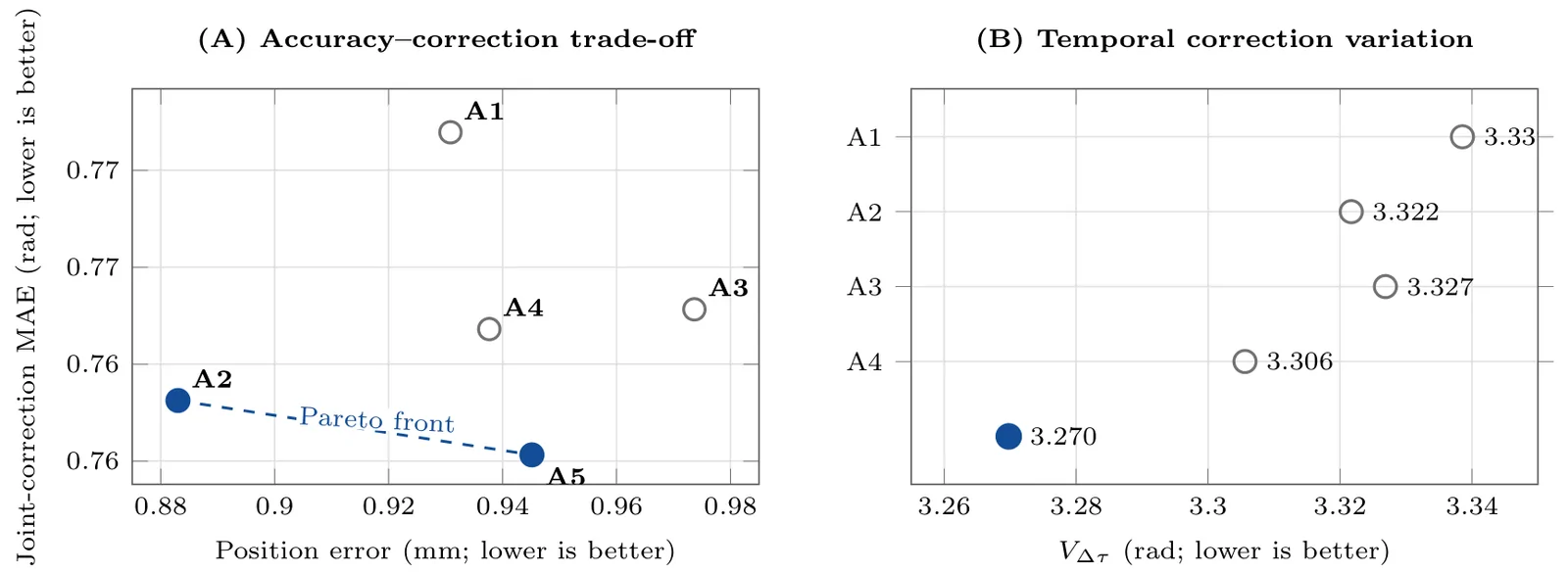
Multi-objective trade-off across the constraint settings. (**A**) Position error versus joint correction MAE; A2 and A5 are the non-dominated settings. (**B**) Mean temporal correction variation, shown separately to avoid a visually ambiguous bubble-sized encoding.

**Figure 8:**
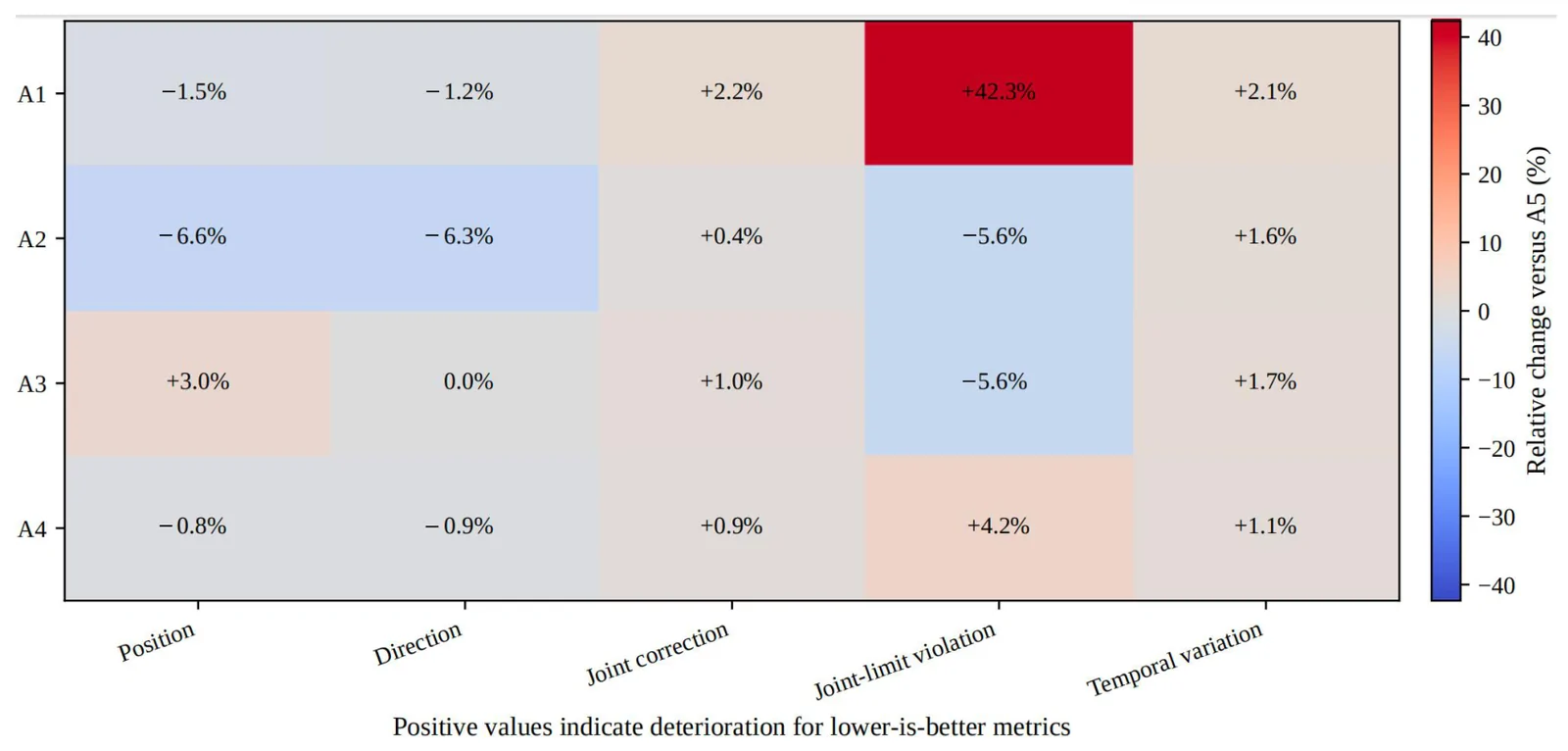
Relative effect of setting each constraint coefficient to zero compared with A5. Positive values indicate deterioration for lower-is-better metrics.

A2 achieved the lowest target position and direction errors, whereas A5 achieved the lowest joint correction MAE and temporal variation. Figure 7 therefore presents the settings as a multi-objective trade-off. Figure 8 summarizes the relative effect of setting each constraint coefficient to zero.

### 3.4. Computational Cost and Reproducibility

The 25 scheduled training runs required a total wall-clock time of 666.91 s, corresponding to an average of 26.68 s per model. For the complete A5 configuration, the mean fold-level training time was 24.22±7.87 s. All runs completed successfully and generated the expected checkpoints, metric files and prediction outputs. The computational reproducibility and workspace feasibility checks are summarized in Table 6. Additional fold-wise results and run integrity checks are provided in the Supplementary Materials File S1.

### 3.5. Workspace Margin Feasibility Audit

For a symmetric axis-aligned clearance, a nonempty feasible interval requires

(15) $$2d_{\mathrm{safe}}<\min_{j}\left(\omega_{\max,j}-\omega_{\min,j}\right).$$

The recorded workspace widths were 76.261, 56.707 and 65.515 mm, whereas the configured clearance was 30 mm per side. Because the resulting total clearance of 60 mm exceeded the narrowest workspace dimension of 56.707 mm, the binary workspace clearance indicator was saturated and was therefore excluded from comparative safety ranking. Equation (15) provides a necessary feasibility check for workspace margin selection before model training and evaluation.

## 4. Discussion

PCD-Net was designed as a front-end target representation module rather than as a replacement for conventional robot planning. It jointly predicts a base-frame target position, principal insertion direction and seven-joint correction vector, while MoveIt2 remains responsible for roll assignment, inverse kinematics, collision checking and trajectory generation. This modular interface restricts the learned component to a clearly defined task while preserving established planning safeguards.

The pipeline-level and fivefold analyses provide complementary evidence. Compared with the unconstrained neural baseline, PCD-Net reduced the direction error while maintaining a comparable planning time. The zero-weight analysis further showed that the joint limit term reduced the violation tendency, whereas correction-magnitude regularization improved the joint correction accuracy and temporal consistency across all held-out target sequences. The complete objective therefore provided a balanced solution between the geometric targeting accuracy and joint-level feasibility rather than minimizing each metric independently.

A2 removed the auxiliary cosine consistency term while retaining supervised direction-vector fitting. Its relatively low direction error indicates that supervised fitting already provided substantial angular guidance, whereas the cosine term acted as an additional unit-direction regularizer. This result supports interpreting the loss terms according to their operational roles rather than assuming that each term must improve every evaluation metric independently.

PCD-Net differs from governing equation-based PINNs because it embeds explicit robot-state, joint limit and workspace constraints into a two-stage dual-branch front end rather than enforcing differential equation residuals. The workspace-margin audit provides an additional implementation safeguard: a symmetric boundary penalty is meaningful only when the requested margin is smaller than half of the narrowest workspace dimension. Applying the feasibility condition in Equation (15) prevents an infeasible parameter setting from being misinterpreted as evidence of safety.

Several limitations remain. All evidence was obtained in simulation, and the public CT-derived models were not clinically characterized DDH cases. The independent cross-validation units were five target sequences rather than patients or pelvises. The network predicts a principal insertion axis rather than a complete end-effector orientation, with the tool roll assigned by the downstream planner. In addition, the current comparison was limited to standard IK and RRTConnect, an unconstrained neural baseline and constraint-reduced variants. Future work should compare constrained inverse-kinematics and optimization-based methods under matched computational budgets. Phantom experiments, physical robot execution, registration uncertainty analysis, tool–tissue interaction modeling and evaluation using clinically stratified DDH anatomy are required before surgical translation.

## 5. Conclusions

This study developed PCD-Net, a physically constrained dual-branch front-end network for DDH-oriented surgical robot navigation. The network jointly predicts a base-frame target position, principal insertion direction and seven-joint correction vector for downstream MoveIt2 and OMPL planning. Pipeline-level experiments demonstrated sub-millimeter position accuracy, sub-degree direction accuracy and millisecond-scale planning, while target sequence-level fivefold analysis showed that the complete constraint setting achieved the lowest joint correction error and temporal correction variation. The joint limit penalty reduced the violation tendency, and the workspace-margin audit established an explicit feasibility condition for safety gap parameter selection. These results support PCD-Net as a computationally lightweight and planner-compatible front-end optimization method. Phantom experiments, physical robot validation and evaluation using clinically characterized DDH anatomy remain necessary before surgical translation.

## Figures and Tables

**Table 1:** Input, output and network specification of PCD-Net.

Component	Specification	Dimension
Input vector	pB (3), dB (3), q (7), $\omega_{\min}$ (3), $\omega_{\max}$ (3), $d_{\mathrm{safe}}$ (1) and γ (1)	21
Preprocessing	Fold-wise feature standardization and insertion-direction normalization	–
Shared trunk	Linear(21,256)–ReLU–Linear(256,256)–ReLU–Linear(256,256)–ReLU	–
Position–direction head	Linear(256,128)–ReLU–Linear(128,64)–ReLU–Linear(64,6)	6
Joint correction head	Linear(256,128)–ReLU–Linear(128,64)–ReLU–Linear(64,7)	7
Network outputs	${\hat{p}}_{B}$ (3), ${\hat{d}}_{B}$ (3) and $\Delta \hat{\tau }$ (7)	13
Trainable parameters	Total number of weights and biases	220,365

**Table 2:** Implementation environment and computational configuration.

Item	Configuration
Operating system	Ubuntu 22.04.5 LTS
Processor and memory	Intel Core i5-12500H and 15 GiB usable system memory
Graphics processor	NVIDIA GeForce RTX 3050 Laptop GPU with 4 GiB memory
Software environment	Python 3.10.12, PyTorch 2.11.0+cu130, CUDA 13.0, ROS2 Humble and Gazebo 11.14.0
Optimization settings	Adam optimizer, learning rate $1\times 10^{-3}$, batch size 64 and random seed 42
Training schedule	120 supervised-pretraining epochs followed by 180 constraint-fine-tuning epochs
Checkpoint selection	Lowest validation total loss during constraint fine-tuning
Computational cost	666.91 s for 25 training runs; 26.68 s per model on average

**Table 3:** Constraint configurations used in the target sequence-level fivefold analysis.

Group	Configuration
A1	Coefficient of $L_{\mathrm{kin}}$ set to zero
A2	Coefficient of $L_{\cos}$ set to zero; supervised direction fitting was retained
A3	Coefficient of $L_{\mathrm{corr}}$ set to zero
A4	Coefficient of $L_{\mathrm{ws}}$ set to zero
A5	Complete PCD-Net objective with all constraint terms enabled

**Table 4:** Pipeline-level descriptive comparison across 15 complete simulation trials. All reported methods completed 15/15 trials successfully.

Method	Position Error (mm)	Direction Error (°)	Planning Time (s)
G1: standard IK + RRTConnect	0.774±0.164	0.441±0.168	0.0179±0.0044
G2: unconstrained neural network	0.682±0.256	0.458±0.148	0.0161±0.0036
G3-NP: dual branch without constraint terms	0.760±0.263	0.457±0.158	0.0192±0.0060
G3-NR: without correction magnitude regularization	0.726±0.269	0.429±0.137	0.0174±0.0063
PCD-Net: complete deployed model	0.736±0.202	0.428±0.130	0.0162±0.0040

**Table 5:** Target sequence-level fivefold results. Values are fold means ± between-fold SD.

Group	Position Error (mm)	Direction Error (°)	Joint Correction MAE (Rad)	Joint Limit Violation Rate	Temporal Correction Variation (Rad)
A1	0.931±0.291	0.230±0.067	0.772±0.041	0.101±0.062	3.339±0.610
A2	0.883±0.214	0.218±0.042	0.758±0.050	0.067±0.042	3.322±0.640
A3	0.974±0.257	0.233±0.066	0.763±0.042	0.067±0.050	3.327±0.647
A4	0.938±0.296	0.231±0.065	0.762±0.041	0.074±0.052	3.306±0.625
A5	0.945±0.287	0.233±0.065	0.755±0.034	0.071±0.049	3.270±0.589

Bold values indicate the lowest (best) result in each column.

**Table 6:** Computational reproducibility and workspace feasibility checks.

Check	Result
Completed training runs	25/25; no failed configurations
Mean training time	26.68 s per model; A5 fold mean 24.22±7.87 s
Narrowest workspace dimension	56.707 mm (Y axis)
Configured symmetric gap	30.000 mm per side
Feasibility condition	$2d_{\mathrm{safe}}<\min_{j}\left(\omega_{\max,j}-\omega_{\min,j}\right)$
Outcome	The requested 60.000 mm total gap exceeded the 56.707 mm narrowest dimension; the saturated clearance indicator was excluded from comparative safety ranking.

## Data Availability

The fold-level numerical results, target sequence partitions and model and training specifications used in this study are included in the Supplementary Materials File S1 and the source data folder is included in the Supplementary Materials File S2. The public anatomical source is available through the TotalSegmentator dataset under its applicable data use terms. Additional simulation configuration files are available from the corresponding author upon reasonable request.

## Supplementary Materials

The following supporting information can be downloaded at https://www.mdpi.com/article/10.3390/bioengineering13080895/s1. File S1. The supporting information includes fold-wise results for the 25 trained models, target sequence partitions, complete model and training specifications, planner interface details, run integrity checks and the workspace-margin feasibility analysis. File S2. source_data.
