# Supplementary material for: Physically Constrained Dual-Branch Front-End Optimization for DDH-Oriented Surgical Robot Navigation
Source: Bioengineering (Basel). 2026 Aug 3;13(8):895. doi: 10.3390/bioengineering13080895 (PMC13509604; doi:10.3390/bioengineering13080895)
Supplement: Supplementary file 1 [file bioengineering-13-00895-s001.zip › File S1. Supplementary_Materials.pdf]

Supplementary Materials

Physically Constrained Dual-Branch Front-End Optimization for DDH-Oriented Surgical Robot Navigation

Jiabao Li et al.

Overview

These supplementary materials provide the fold-wise component results, the complete target sequence partitions, the exact model and training specifications and the workspace-margin feasibility audit supporting the manuscript. The cross-validation unit was a complete simulated target sequence; individual frames from the same sequence were never split across training, validation and testing within a fold.

Table S1. Fold-Wise Results of the 25 Independently Configured Models

Fold-wise held-out-sequence results. The within-sequence SD columns describe frame-level variability within the held-out sequence and are distinct from the between-fold SD reported in the main manuscript.

Fold	Group	Position error (mm)	Within-seq. SD (mm)	Direction error (°)	Joint correction MAE (rad)
1	A1	1.427919	0.749867	0.341180	0.739547
1	A2	1.200460	0.566462	0.279224	0.710984
1	A3	1.419688	0.746411	0.339533	0.741018
1	A4	1.426661	0.748659	0.339655	0.740540
1	A5	1.426232	–	0.339841	0.740448
2	A1	0.893168	0.618125	0.176892	0.767890
2	A2	0.943882	0.669587	0.179878	0.732396
2	A3	0.931607	0.665878	0.179137	0.731117
2	A4	0.957120	0.677400	0.178199	0.731434
2	A5	0.955554	0.679092	0.180163	0.728302
3	A1	0.758508	0.616214	0.178957	0.725931
3	A2	0.746992	0.615197	0.177196	0.724877
3	A3	0.761221	0.611775	0.176985	0.725640
3	A4	0.758033	0.612237	0.180604	0.724690
3	A5	0.758025	0.612374	0.180041	0.724724
4	A1	0.689675	0.382245	0.223944	0.824553
4	A2	0.637109	0.370970	0.227751	0.820765
4	A3	0.870116	0.364832	0.238543	0.814511
4	A4	0.660238	0.379246	0.226733	0.810975
4	A5	0.699708	0.386639	0.235462	0.781723
5	A1	0.884980	0.561273	0.230090	0.801875
5	A2	0.886686	0.561353	0.227511	0.801596
5	A3	0.885680	0.563134	0.230836	0.801848
5	A4	0.886280	0.561574	0.230232	0.801408
5	A5	0.886216	0.561519	0.230013	0.801408

Note: The within-sequence SD for Fold 1 A5 was not available in the stored metric record and is shown as an em dash; no value was imputed.

Table S2. Target Sequence-Level Fivefold Partitions

Fold	Training Sequences	Validation	Testing	Train Rows	Val. Rows	Test Rows
1	T03, T04, T05	T02	T01	600	199	200
2	T01, T04, T05	T03	T02	600	200	199
3	T01, T02, T05	T04	T03	599	200	200
4	T01, T02, T03	T05	T04	599	200	200
5	T02, T03, T04	T01	T05	599	200	200

The complete sequence, rather than the individual frame, was the unit of partitioning. This prevents neighboring frames from the same simulated target trajectory from appearing in both the model development and test subsets.

Table S3. Complete Model, Loss and Training Specification

Item	Specification
Input dimension	21
Target geometry	Robot base frame target position (3) and unit insertion direction (3)
Robot state	Seven current Panda joint variables
Workspace input	Lower and upper axis-aligned workspace bounds (6)
Constraint scalars	Symmetric gap parameter d_{safe} and correction regularization factor γ
Shared trunk	Linear(21,256)–ReLU–Linear(256,256)–ReLU–Linear(256,256)–ReLU
Position–direction head	Linear(256,128)–Linear(128,64)–Linear(64,6)
Joint correction head	Linear(256,128)–Linear(128,64)–Linear(64,7)
Trainable parameters	220,365
Position–direction output	Base-frame position (3) and principal insertion-direction vector (3)
Joint correction output	Seven-dimensional $\Delta\boldsymbol{\tau} = \mathbf{q}_{\text{goal}} - \mathbf{q}_{\text{current}}$
Pretraining	120 epochs, supervised position, direction and joint correction fitting
Constraint fine-tuning	180 epochs
Optimizer	Adam
Learning rate / batch size	10^{-3} / 64
Random seed	42
Checkpoint rule	Lowest validation total loss during the constraint fine-tuning stage
λ_{pos}	1.0
$\lambda_{\text{dir,sup}}$	0.2
λ_{τ}	1.0
λ_{cos}	5.0
λ_{kin}	0.1
λ_{corr}	0.01
λ_{ws}	1.0
Hardware	Intel Core i5-12500H, 15 GiB RAM, NVIDIA RTX 3050 Laptop GPU (4 GiB)
Software	Ubuntu 22.04.5 LTS; Python 3.10.12; PyTorch 2.11.0+cu130; CUDA 13.0; ROS2 Humble; Gazebo 11.14.0

Algorithm S1. Two-Stage Target Sequence-Level Model Evaluation

1. Group all frames by complete target sequence identifier T01–T05.
2. For each fold, assign three complete sequences to training, with one to validation and one to testing, as specified in Table S2.

3. Standardize input and supervised target variables using training sequence statistics only.
4. Train the shared trunk and both output heads for 120 supervised epochs.
5. Continue training for 180 epochs using the configured constraint setting A1–A5.
6. Select the checkpoint with the lowest validation total loss during constraint fine-tuning.
7. Evaluate the selected checkpoint once on the held-out complete target sequence.
8. Store the held-out predictions, configuration and metric record; then, audit the expected files and compare the within-fold prediction outputs for duplication.

Algorithm S2. Planner-Side Label Generation and Inference Interface

1. Transform the anatomical target point and insertion direction into the robot base frame.
2. Construct the planner-side Cartesian goal using the transformed position, principal tool axis and the fixed roll convention.
3. Use MoveIt2 to solve inverse kinematics under the common joint limits and collision model; denote a successful solution by $\mathbf{q}_{\text{goal}}$.
4. For successful planning records, define the supervised correction label as $\Delta\boldsymbol{\tau} = \mathbf{q}_{\text{goal}} - \mathbf{q}_{\text{current}}$.
5. During inference, provide $\hat{\mathbf{p}}_B$ and $\hat{\mathbf{d}}_B$ as the Cartesian goal representation and $\mathbf{q}_{\text{current}} + \Delta\hat{\boldsymbol{\tau}}$ as a feasibility-conditioned joint proposal.
6. Retain MoveIt2 or OMPL for roll assignment, inverse kinematics, collision checking and RRTConnect trajectory generation. Records with unsuccessful planning are excluded before sequence partitioning; no loss-based filtering is applied afterward.

Note S1. Workspace-Margin Feasibility Analysis

For an axis-aligned workspace $[\boldsymbol{\omega}_{\min}, \boldsymbol{\omega}_{\max}]$ and a symmetric margin d_{safe}, a nonempty feasible interval exists only when

$$2d_{\text{safe}} < \min_j \left(\omega_{\max,j} - \omega_{\min,j}\right). \tag{1}$$

The retained workspace widths were 76.261 mm, 56.707 mm and 65.515 mm for the X, Y and Z axes, respectively. The configured gap was 30.000 mm per side, requiring a total of 60.000 mm. Because 60.000 mm exceeded the narrowest width of 56.707 mm, the requested symmetric interior region was empty along the Y axis. The resulting binary clearance indicator saturated across all settings and was therefore excluded from comparative safety claims. The implementation treats the inequality above as a compulsory parameter-validity check before model training or evaluation.

Reproducibility Audit Summary

- Planned model configurations: 25; accepted model outputs: 25; failed configurations: 0.
- Pairwise prediction file comparisons performed within the five folds: 50; duplicate pairs: 0.
- Total recorded batch wall-clock time: 666.91 s; mean time per configured model: 26.68 s.
- The reported evidence is at the simulation level and target sequence level; it is not at the patient level or on a clinical validation dataset.
